# Effect of carbonate apatite as a bone substitute on oral mucosal healing in a rat extraction socket: in vitro and in vivo analyses using carbonate apatite

**DOI:** 10.1186/s40729-022-00408-4

**Published:** 2022-03-07

**Authors:** Yuki Egashira, Ikiru Atsuta, Ikue Narimatsu, Xiaoxu Zhang, Ryosuke Takahashi, Kiyoshi Koyano, Yasunori Ayukawa

**Affiliations:** 1 Section of Implant and Rehabilitative Dentistry, Division of Oral Rehabilitation, Faculty of Dental Science, Kyushu University, Fukuoka, Japan; 2 Division of Advanced Dental Devices and Therapeutics, Faculty of Dental Science, Kyushu University, 3-1-1 Maidashi, Higashi-ku, Fukuoka, 812-8582 Japan

**Keywords:** Carbonate apatite, Epithelial seal, Oral mucosa, Adhesion molecule, Animal model

## Abstract

**Background:**

Low bone quantity and quality are serious problems that affect the prognosis of implants in the cosmetic field. Therefore, artificial bone substitutes are frequently used. However, whether there is a difference in the effect of either bone substitute on soft tissue healing is unclear given their greatly different absorbability. In this study, we used hydroxyapatite (HAp) and carbonate apatite (CO_3_Ap) as bone substitutes to analyze the epithelial and connective tissue healing after tooth extraction.

**Methods:**

In vitro, oral mucosa-derived epithelial cells (OECs) collected from 4-day-old Wistar rats were seeded on HAp or CO_3_Ap and evaluated for adhesion, proliferation, migration, apoptosis, and morphology. Fibroblasts (FBs) were also analyzed for their ability to express collagen. In vivo, the extraction of maxillary right first (M1) and second molars (M2) of 6-week-old male Wistar rats was performed, followed by insertion of HAp or CO_3_Ap granules into the M1 and M2 sites. The oral mucosal healing process was then evaluated histochemically after 7 and 14 days.

**Results:**

In vitro, high collagen expression by FBs in the CO_3_Ap group was observed and the surface analysis showed spreading of the FBs on the CO_3_Ap surface. However, the activity of OECs was suppressed on CO_3_Ap. Two weeks after CO_3_Ap implantation, soft tissue healing was observed, and recovery of the connective tissue was observed on the remaining CO_3_Ap.

**Conclusions:**

Our results suggest that the formation of soft tissues, including connective tissue, was promoted by CO_3_Ap in the extraction socket within a short period.

## Background

The methods and techniques of implant treatments have improved dramatically since the 1950s, and the range of clinical adaptation has expanded significantly [1, 2]. These developments are supported by bone augmentation with bone substitutes, which has become an indispensable treatment option as a pretreatment for implant treatment [3]. In addition, there are many bone substitutes, and they are used based on the preference and experience of the operator, as well as the operator’s understanding of the characteristics of the material.

However, these choices are often based on the bone-retaining characteristics of bone substitutes, such as the affinity and absorbability, and their influence on surrounding tissues, especially soft tissues, may not be sufficiently considered. Indeed, understanding soft tissue response and controlling the post-treatment morphology enhances the esthetics, cleanliness, and sometimes functionality of the implant treatment [4, 5]. Therefore, in this experiment, we evaluated the healing process of the soft tissue covering the bone substitutes and considered how it is affected by the implanted materials.

In this study, a bone substitute was placed in the extraction socket of a rat and the soft tissue healing over the materials was observed over time. At the first step in the healing of soft tissues, once the bleeding occurs because of the vascular injury, platelets accumulate, vasoconstriction stops the bleeding, and macrophages phagocytose the necrotic tissue. The next step is the proliferative phase. During this period, collagen-based granulation tissue secreted by fibroblasts is formed. Finally, epithelial cells form the basement membrane on the connective tissue repaired by the collagen, and the epithelial tissue is further formed as a layer [6]. Such healing steps occur directly above the bone substitute after filling. Specifically, when bone augmentation using a bone substitute is performed, the material is in direct contact with the connective tissue when a biological membrane is not used. However, when a membrane is used, it is can indirectly affect the connective tissue and epithelial tissue. Therefore, differences in the material may affect the healing speed and mucosal morphology.

Many types of bone substitute have been used [7, 8]. Among them, autologous bone is the gold standard [9]; however, artificial materials have been widely clinically applied in recent years in consideration of their low invasiveness to patients [10]. In this study, hydroxyapatite (HAp), which has been clinically used for a long time, and carbonate apatite (CO_3_Ap), which has the same composition as the inorganic component of bone, were used. We evaluated the reaction of soft tissues in both in vitro culture experiments and animal experiments.

In this study, we evaluated the effects of these bone substitutes on the dynamics of epithelial cells and fibroblasts. Furthermore, the speed of wound healing as a macroscopic finding and the morphology of the soft tissue histologically were evaluated. The above experimental techniques were previously performed in our groups [11], and we considered them to be highly credible and acceptable as experimental methods.

This result will be noteworthy when considering the dehiscence and scarring of the wound over a bone substitute, and the inhibition of bone replacement by soft tissue. The results of this paper will provide a new selection criterion for many materials, enabling the selection of a more appropriate bone substitute for each case, which will be useful for predicting the prognosis.

## Methods

### Epithelial cell and fibroblast culture

**Fig. 1:**
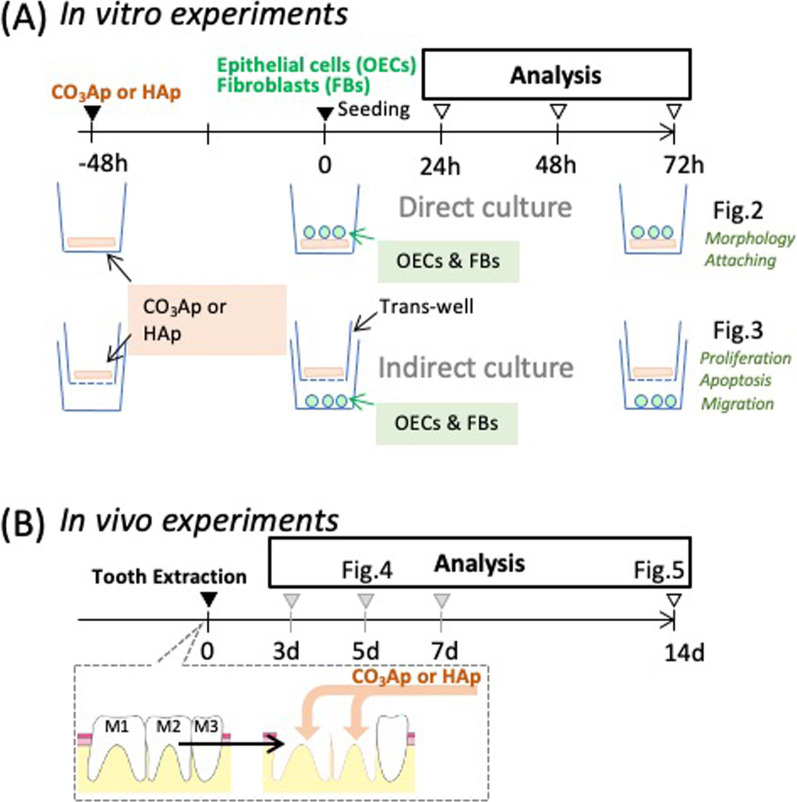
In vivo and in vitro experimental design. **A** The experimental protocol of the in vitro study. The upper stage showed that OECs or FBs were cultured directly on the material (CO_3_Ap or HAp). In the lower stage, the material was placed in the Transwell and each cell was indirectly cultured. **B** The experimental protocol for the in vivo study. Bone substitute (CO_3_Ap or HAp) filling was performed immediately after tooth extraction (M1 and M2) and the healing of soft tissues was observed over time

Rat oral epithelial cells (OECs) and fibroblasts (FBs) cultures were established as previously described [11] (Fig. 1A). Briefly, these cells were individually isolated from oral mucosa from 4-day-old Wistar rats. Mucosal OECs were cultured in defined keratinocyte serum-free medium (DK-SFM; Invitrogen, Grand Island, NY, USA), and FBs from the connective tissue were grown in minimal essential medium (MEM; Invitrogen) containing 10% fetal bovine serum (FBS; Life Technologies, Carlsbad, CA, USA). This experiment followed the guidelines established by Kyushu University (approval number: A29-227-0).

### Scanning electron microscopy observation

**Fig. 2:**
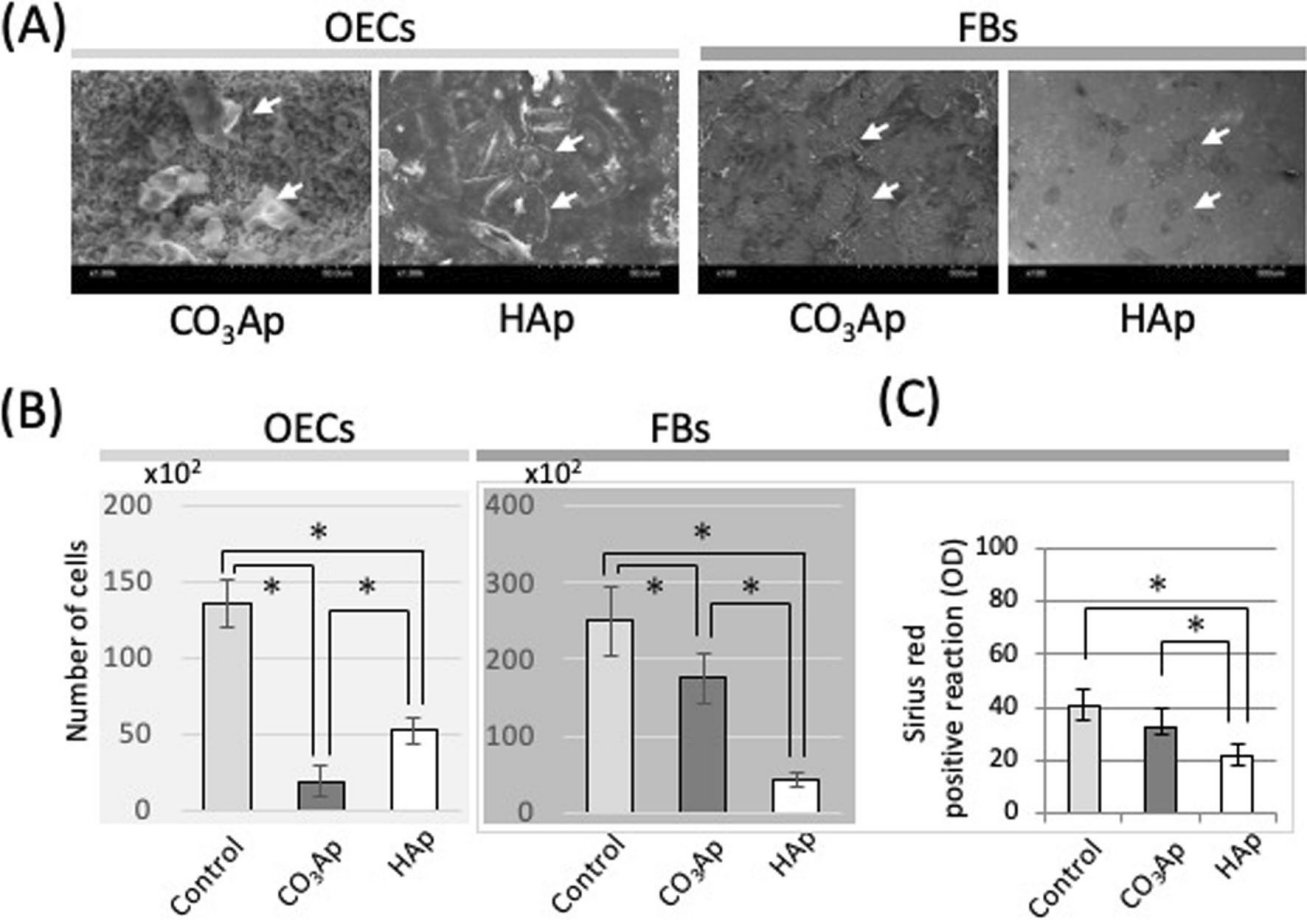
Direct effects of carbonate apatite on OECs and FBs. **A** Scanning electron microscopy images of OECs or FBs on the materials. **B** The number of cells on the materials (n = 6, *p < 0.05). **C** Quantification of Sirius red staining for collagen expression of FBs on the materials. Control: culture dish, CO_3_Ap: carbonate apatite disc, HAp: hydroxyapatite disc

The OECs and FBs morphology on CO_3_Ap or HAp plates (diameter 10.0 mm, thickness 0.8 mm), which was kindly provided by GC (Tokyo, Japan), was evaluated by scanning electron microscopy (SEM) [12] (Fig. 2A). All samples were fixed with 2.5% glutaraldehyde, dehydrated by graded ethanol solutions, and then freeze-dried. Samples were mounted on stubs, coated with an Au/Pd alloy, and evaluated microscopically.

### Evaluation of OEC and FB adhesion

Adhesion assays were performed following methods described in our previous report. Briefly, non-adherent cells were removed by shaking the specimens using a rotary shaker (NX-20; Nissin, Tokyo, Japan), and adherent cells were counted.

### Collagen production from FB

To quantify collagen production, a colorimetric assay based on Sirius red staining was performed after culture with CO_3_Ap and HAp plates or on culture dishes. Samples were washed with PBS and treated with 0.2% aqueous phosphomolybdic acid, then stained with Sirius red dye (Wako Pure Chemical Industries, Osaka, Japan) dissolved in saturated aqueous picric acid (pH 2.0). The samples were then treated for 30 min with 0.1 N sodium hydroxide to de-stain and release bound dye into the solution. The optimal density (OD) of this solution was then identified using a spectrophotometer at 550 nm, with 0.1 N sodium hydroxide as the blank.

### Transwell analyses

**Fig. 3:**
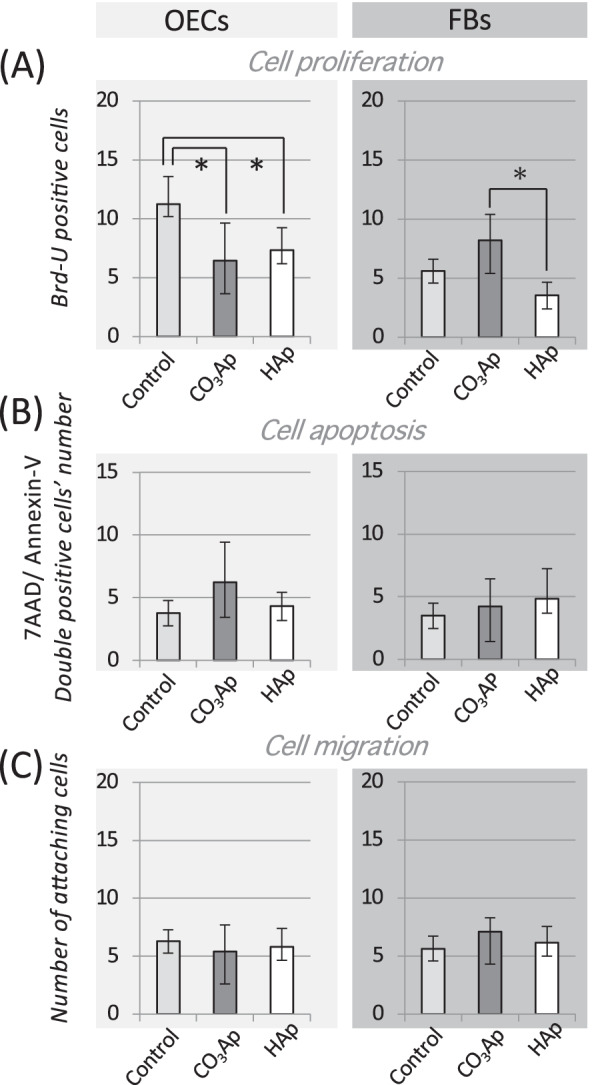
Indirect effects of carbonate apatite on OECs and FBs. **A** BrdU assays for cell-proliferation estimation. **B** FACs assays for cell apoptosis estimation. The number of double-positive 7AAD and Annexin-V cells were counted. **C** Migration assay. The number of cells migrated from the edge of the wound was counted (n = 6, *p < 0.05)

Cells were co-cultured indirectly with materials using a Transwell® insert as a separator. Briefly, OECs and FBs were cultured on a culture well and then Transwell inserts with or without materials (CO_3_Ap and HAp) in the upper chamber served as experimental or control groups (Fig. 3) for the various assays described below.

### Proliferation analyses

OECs or FBs were cultured in the bottom chamber on a Transwell system. As shown in Fig. 1A, the upper chamber contained CO_3_Ap or HAp. Cell proliferation was detected using a Cell Proliferation Kit (GE Healthcare). Briefly, OECs or FBs were exposed to 5-bromo-2′-deoxyuridine (BrdU) in culture medium for 24 h, fixed in 70% methanol for 30 min, and incubated with an anti-BrdU antibody for 1 h. The cells were then incubated with FITC-conjugated anti-mouse IgG (Thermo Fisher Scientific; 1:100) and counted.

### Fluorescence-activated cell sorting (FACS) analyses

For apoptosis analyses, OECs or FBs on CO_3_Ap, HAp plates, or cultures dishes were incubated with Annexin-V-FITC and 7AAD-peridinin chlorophyll protein (Apoptosis Detection Kit; BD Biosciences Ltd., Franklin Lakes, NJ, USA). The analysis was carried out using a FACSCalibur system (BD Biosciences Ltd.) [13].

### Migration assays

As previously described [14], confluent monolayers of OECs or FBs were wounded with a cell scraper and cultured for 48 h. OECs or FBs at the edge of the wound were observed by immunofluorescence using antibodies against actin for cell visualization.

### Animals

Rats received care following the guidelines established by Kyushu University (approval number: A29-227-0). Extraction and transplant was performed as previously reported [12]. Briefly, 6-week-old Wistar rats (72 males; 120–150 g) had extraction of the maxillary right first and second molars under systemic anesthesia. The extraction socket was enlarged with a dental round bar, and the CO_3_Ap (Cytrans Granule, GC; particle size 0.3–0.6 mm) or HAp bone substitute (Bonetite granule perio, HOYA Technosurgical, Tokyo Japan; particle size 0.3–1.0 mm) was implanted [12].

### Micro-CT

**Fig. 4:**
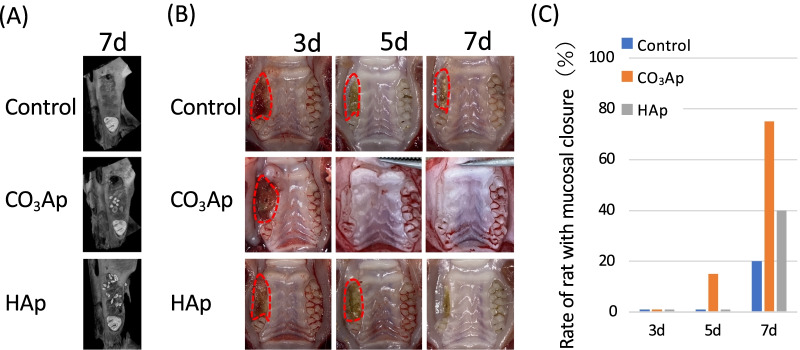
Effect of carbonate apatite on soft tissue healing. **A** Image of the horizontal section at the observation site (schematic and CT image). **B** Soft tissue closure was observed macroscopically and over time on intra oral photographs after tooth extraction and material filling. **C** Percentage of mucosal closure (n = 6)

After the rats were killed, their maxillae were corrected, and fixed in 10% paraformaldehyde (Merck, Darmstadt, Germany) for 24 h. Micro-CT imaging was performed (SkyScan 1076; Bruker micro-CT; tube current: 201 μA; voltage: 49 kV; pixel size: 18 μm) and three-dimensional analysis software (CTAn, Bruker micro-CT) was used for analysis (Fig. 4A).

### Wound healing

The length of distance between the edges of the epithelial surfaces was calculated for three sections of the maxillae, namely the midsection of the extraction socket and sections 100 μm mesial and distal to the midsection. All measurements were done three times and the average calculated.

### Histochemistry with light microscopy

**Fig. 5:**
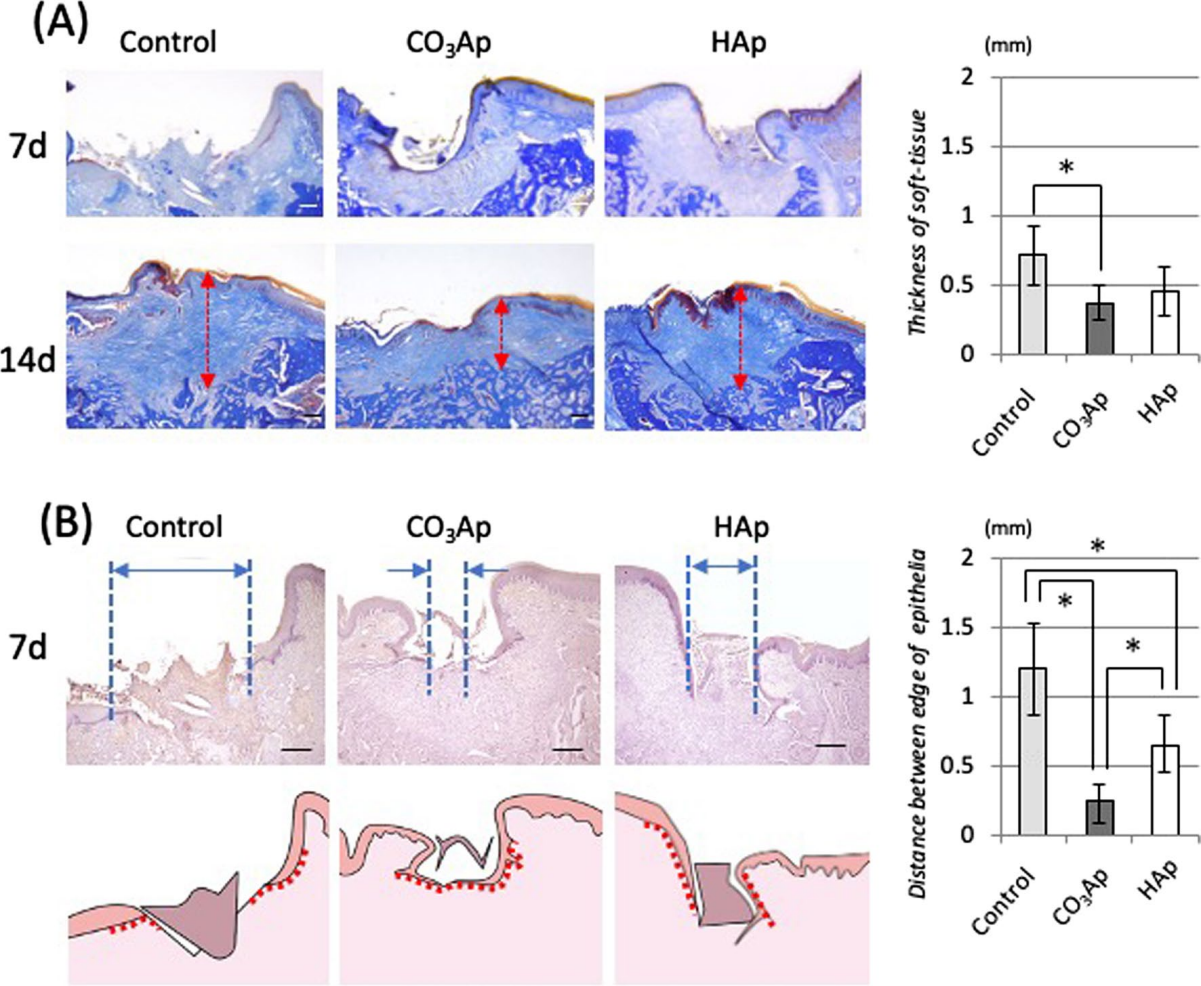
Soft tissue structures on the healed tooth extraction sockets filled with bone substitute. **A** Ladewig’s fibrin staining of the covered soft tissue structure. **B** Immuno-stained histology with Laminin-332. The length of distance between the edges of the epithelial surfaces

The oral mucosa from the rat was cut into sections on the coronal plane using a cryostat. For immuno-histochemical staining, these sections were stained with rabbit anti-Ln-332 (Chemicon International Inc., Temecula, CA, USA), biotinylated anti-rabbit IgG (Sigma, St. Louis, MO, USA), and visualized by a diaminobenzidine (DAB) staining kit (Vector Laboratories, Burlingame, CA, USA) as previously reported [13]. Some sections were stained with Ladewig’s fibrin stain to observe the connective tissue (Fig. 5A).

### Statistical analysis

Our experiment used six samples in each group, and an a priori Shapiro–Wilk test was performed to test for normality. One-way analysis of variance (ANOVA) with Scheffe’s post hoc was performed. Values of *p* < 0.05 were considered statistically significant. Data are indicated as the mean ± standard deviation (SD).

## Results

### Cell dynamics on carbonate apatite

As shown in the top row of Fig. 1A, OECs and FBs were directly cultured on CO_3_Ap or HAp. As shown in the SEM image, the OECs were spheroidized in the CO_3_Ap group, whereas the cells were elongated on HAp, showing a strong adhesive shape unique to OECs. Alternatively, the FBs data were the opposite. FBs were more elongated on the surface irregularities of CO_3_Ap than HAp (Fig. 2A). As shown in Fig. 2B, the number of adherent OECs and FBs on both materials was lower than on the Control group. However, the number of FBs adhered to the CO_3_Ap adhered was significantly greater than on the HAp. Furthermore, the collagen expression capacity on HAp was significantly less than the CO_3_Ap group, and there was no difference between the Control group and CO_3_Ap group (Fig. 2C).

### Indirect effects of carbonate apatite on cells

As shown in the bottom row of Fig. 1A, OECs and FBs were cultured under a non-contact situation using a Transwell. The cell-proliferation ability was observed using Brd-U assay, as shown in Fig. 3A. OECs showed a decrease in Brd-U-positive cells in the presence of material compared with the Control. Alternatively, FBs showed a clear increase on the CO_3_Ap group compared with the HAp groups.

The effect on apoptosis was clearly greater in OECs in the CO_3_Ap group, though not significantly in others (Fig. 3B). Cell migration was assessed by the number of cells that moved into wound area (bottom of Fig. 1A); however, neither OECs nor FBs were significantly affected by the materials.

### Effect of carbonated apatite on the soft tissue healing

Using the model shown in Fig. 1B, the effect of CO_3_Ap on soft tissues was evaluated by the extraction socket healing rate. Figure 4A shows a micro-CT image 7 days after filling, and some granules of the bone substitute were observed at the extraction socket centering on M2. In the Control group without any materials in the extraction socket, there was soft tissue dehiscence even after 7 days (Fig. 4B). However, more than 60% of the CO_3_Ap group had wound closure (Fig. 4C). Alternatively, dehiscence was also observed in HAp, and some individual animals were delayed compared with the Control.

### Morphological comparison of the healed soft tissue

Two weeks after implanting the substitute, soft tissue closure was completed in all three groups. Dermal thickening was prominent in the Control groups, and collagen fibers were irregular in the connective tissue. Alternatively, the soft tissue in the CO_3_Ap group had a normal, mucosa-like structure. Specifically, the epidermis had epithelial papillae at regular intervals, and the connective tissue showed a regular structure parallel to the bone surface.

One week after implanting the substitute, the immuno-histological evaluation using Laminin-332 is shown in Fig. 5B. The laminin-positive area was not observed at the boundary between the epidermis and dermis in the Control and HAp groups; however, its expression was observed in the basement membrane in the CO_3_Ap group. The distance between the edges of the epithelial surfaces was significantly shorter in the CO_3_Ap group groups than in the Control and HAp groups.

## Discussion

Bone substitutes assists bone formation at a bone defect that otherwise cannot be cured by self-renewal. The focus of research has been on how efficiently a substitute material can be replaced with high-quality bone and how long it can be maintained [15–17]. However, when an artificial material is used as a bone substitute, it is sometimes recognized as a foreign substance and can affect the bone tissue and other tissues of the body [18]. In this study, we focused on the effects on soft tissues, especially in the early closure stage, and compared carbonate apatite with commonly used HAp.

Bone substitutes rarely come into direct contact with soft tissue because they are typically covered with a membrane or similar after implantation [15]. However, membranes are not used in small wounds, such as “socket preservation” [19], and it is experimentally important to evaluate the direct effect of a bone substitute on local soft tissues. As shown in Fig. 2, the dynamics of OECs and FBs on the bone substitute were observed, and the number of adhered cells was counted. The activity of OECs on CO_3_Ap was clearly less than that in HAp. As shown in Fig. 2A, the surface of CO_3_Ap was rougher than that of HAp. The spheroidization of OECs (Fig. 2A) and a decrease in adhesiveness was correlated to the CO_3_Ap substitute surface (Fig. 2B), which is corroborated by a separate study indicating that the activity of OECs is reduced on titanium with a rough surface [13]. Alternatively, FBs have a high activity on rough surfaces [11]. Therefore, the CO_3_Ap group showed high fibroblast adhesion and collagen expression (Fig. 2B, C).

As shown in Fig. 3, a Transwell system was used to evaluate the dynamics of cells in a non-contact state with the bone substitute material, which is similar to the clinical use of membranes. In the CO_3_Ap group, the proliferative ability of the OECs was reduced and the apoptosis was increased. Alternatively, the proliferative capacity of FBs was reinforced and the cells were activated. HAp did not have such an effect, indicating the response was a function of the CO_3_Ap material. Past reports have also shown the release of calcium ions from carbonate apatite in solution [20, 21]. While many papers report that calcium ions increase the adhesion of OECs, Matsui et al. reported that a high concentration of calcium ions reduced the proliferation of OECs and induced keratinocyte death [22], and Sugimoto et al. indicated that calcium ions increased activation of FBs under this culture condition [23]. These reports could explain the effect of materials on OECs and FBs. However, there is also a report that calcium ions are precipitated in HAp [21, 24], indicating that factors other than calcium ions also have an effect.

In vivo experiments were also conducted in a rat maxillary extraction model. The bone substitutes were inserted immediately after tooth extraction, and hemostasis was performed by compression. Various models were developed such as covering with a mucous membrane, fixing with an adhesive, and binding with a suture for mucosa over the extraction socket. However, sutures and adhesives interfered with the evaluation of soft tissue in this experiment. As shown in Fig. 4A, a certain amount of bone filling material remains in the extraction socket, and its influence is sufficiently exerted on the surroundings. The model is considered to be valid because there is a large difference in the alveolar bone formation (data not shown).

As shown in Fig. 4B and C, the soft tissue was clearly promoted to close in the CO_3_Ap group as compared with the Control group without any materials in the socket and HAp group. As shown in the in vitro experiment, each material might promote connective tissue healing via the FBs.

Figure 5 shows the histological image 2 weeks after filling. Ladewig's fibrin staining in Fig. 5A was used to observe the state of connective tissue. The soft tissue of wound healing causes scarring because of hyperplasia of the connective tissue [25]. However, in the CO_3_Ap group, thickening was clearly suppressed as compared with the control groups. Furthermore, the collagen fibers constituting connective tissue was parallel to the bone surface and the density was high. The epithelial basement membrane structure indicated by the Laminin-332 positive area was also restored in the CO_3_Ap group (Fig. 5B). Laminin-332 is an adhesion-related protein expressed by OECs, and it is a component of the basement membrane located at the boundary between the connective tissue dermis and epidermis comprising OECs [26–28]. The normal mucosal structure was nearly restored as indicated by the deposition of this protein. Additionally, heparan sulfate proteoglycan is one of the most important factors for adhesion between the epidermis and dermis [29]. Additionally, the high affinity of heparan/heparan sulfate requires Ca ions at physiological concentrations [30]. Therefore, our results reflect the high wound-healing ability and expression of laminin-332 on CO_3_Ap compared with the other groups. Therefore, the normal condition was restored first because the healing was fast in the CO_3_Ap group. However, previous reports have shown that connective tissue thickening is maintained 4 weeks after the extraction [31], and the difference in the healing rate at 1 week does not affect this result.

CO_3_Ap acts on FBs rather than epithelial tissues, expressing collagen fibers and significantly promoting healing of connective tissue. Mucosal healing proceeds in the following order: (1) expression of collagen fibers by the FBs; (2) wound closure by the connective tissue; (3) expression of laminin by the OECs; (4) formation of the basement membrane; (5) migration and adhesion of OECs on the basement membrane, and (6) epithelial wound closure [32, 33]. Therefore, we believe that the early closure of connective tissue by CO_3_Ap contributes to promoting healing of the entire soft tissue.

However, scarring over the extraction socket proceeds if FBs in the connective tissue are only proliferating, resulting in a regular structure of collagen fibers around the carbonate apatite. Our future work will evaluate why the connective tissue structure was controlled.

## Conclusion

Carbonated apatite applied to an extraction socket promoted soft tissue healing by accelerating wound closure with connective tissue. This suggests that the clinical use of carbonate apatite as a bone substitute was expected to form bone and provide early healing of soft tissues.

## Data Availability

The data used in this study are available from the corresponding author on reasonable request.

## Abbreviations

HAp: Hydroxyapatite
CO_3_Ap: Carbonate apatite
M1: Maxillary right first molar
M2: Maxillary right second molar
BrdU: 5-Bromo-2′-deoxyuridine
OEC: Rat oral epithelial cell
SEM: Scanning electron microscopy
OD: Optimal density
FACS: Fluorescence-activated cell sorting
FBS: Fetal bovine serum
